# Mitochondrial STAT3 regulates antioxidant gene expression through complex I‐derived NAD in triple negative breast cancer

**DOI:** 10.1002/1878-0261.12928

**Published:** 2021-04-10

**Authors:** Tanaya Lahiri, Lara Brambilla, Joshua Andrade, Manor Askenazi, Beatrix Ueberheide, David E. Levy

**Affiliations:** ^1^ Department of Pathology and NYU Perlmutter Cancer Center NYU School of Medicine New York NY USA; ^2^ Department of Biochemistry and Molecular Pharmacology NYU Perlmutter Cancer Center NYU Langone Health Proteomics Laboratory Division of Advanced Research Technologies NYU School of Medicine New York NY USA; ^3^ Biomedical Hosting LLC Arlington MA USA

**Keywords:** breast cancer, glutathione, mitochondria, oxidative stress, reactive oxygen species, STAT3

## Abstract

Signal transducer and activator of transcription 3 (STAT3) is a transcription factor with roles in inflammation and tumorigenicity. A fraction of STAT3 localizes in mitochondria, where it augments tumorigenesis via regulation of mitochondrial functions, including modulation of respiration and redox status. We show a novel mechanism for mitochondrial STAT3 regulation of redox homeostasis in triple‐negative breast cancer cells. Loss of STAT3 diminished complex I dehydrogenase activity and impaired NAD+ regeneration, leading to impaired expression of glutathione biosynthetic genes and other antioxidant genes. Expressing mitochondrially restricted STAT3 or replenishment of the cellular NAD pool restored antioxidant gene expression, as did complementation of the NADH dehydrogenase activity by expression of the STAT3‐independent yeast dehydrogenase, NDI1. These NAD‐regulated processes contributed to malignant phenotypes by promoting clonal cell growth and migration. Proximity interaction and protein pull‐down assays identified three components of complex I that associated with mitochondrial STAT3, providing a potential mechanistic basis for how mitochondrial STAT3 affects complex I activity. Our data document a novel mechanism through which mitochondrial STAT3 indirectly controls antioxidant gene regulation through a retrograde NAD+ signal that is modulated by complex I dehydrogenase activity.

## Graphical abstract

STAT3 interacts with respiratory complex I in mitochondria, leading to enhanced NADH dehydrogenase activity, facilitating efficient regeneration of NAD^+^ during respiration. NAD^+^ acts as a retrograde signal linking mitochondrial metabolism to changes in nuclear gene expression, leading to induction of antioxidant genes that contribute to the maintenance of redox balance and malignant cell growth, survival, and migration.

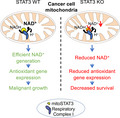

**Abbreviations**
GSH: glutathione
IDH: isocitrate dehydrogenase
KO: knockout
MTS: mitochondrial targeting sequence
NAD(H): nicotinamide adenine dinucleotide
PGC1α: peroxisome proliferator‐activated receptor‐gamma co‐activator‐1alpha
ROS: reactive oxygen species
SIRT: sirtuin
SOD: superoxide dismutase
STAT3: signal transducer and activator of transcription 3
TNBC: triple negative breast cancer
WT: wild‐type

## 1 Introduction

Cellular redox homeostasis is an essential prerequisite for growth and survival of aerobic organisms and is regulated by numerous biochemical pathways that balance production and elimination of reactive oxygen species (ROS). ROS levels are elevated in cancer, where they contribute to DNA damage, proliferation, and metastasis [1]. Although elevated ROS levels are considered drivers of tumorigenesis, they can also trigger apoptosis, senescence, or catastrophic DNA damage; therefore, antioxidant pathways that buffer ROS are necessary for cell survival [2]. A major antioxidant pathway operating in many cancers involves enzymes that detoxify ROS, many of which are products of genes regulated by the NRF2 transcription factor [3], including enzymes involved in the synthesis of the major cellular redox buffer, glutathione (GSH) [4], and enzymes that directly detoxify oxidative environments [5, 6]. Mitochondria are a major source of ROS in tumor cells, with respiratory complex I and III being major contributors [7]. For instance, reduced complex I activity can increase cellular ROS. Mitochondrial complex I is also a major site for generating the cofactor nicotinamide adenine dinucleotide (NAD+) through NADH oxidation, and NAD+/NADH balance maintained by complex I plays a critical role in tumor progression [8]. The matrix arm of complex I, consisting of seven core subunits, binds multiple cofactors including a flavin mononucleotide (FMN) molecule. NADH donates a pair of electrons to this FMN molecule, which then flows through the respiratory chain to ubiquinone. This transfer of electrons at the Fe‐sulfur center N1 can generate ROS. *In vitro* characterization of purified mitochondria shows a strong correlation between complex I‐dependent superoxide production and NAD+/NADH ratio [9, 10]. For example, addition of NADH to purified bovine heart mitochondria generates superoxide, and this phenomenon can be quenched by addition of excess NAD+ [11]. Imbalance in the cellular NAD pool increases disease progression through ROS production and inflammation [12]. NAD+/NADH ratio can also affect gene transcription, largely through the action of the histone deacetylase silent mating type information regulation homolog 1 (SIRT1), which targets chromatin in a NAD+‐dependent manner [13]. SIRT1 target genes help maintain the cellular antioxidant capacity via synthesis of GSH [14] and can contribute to cell survival [15]. SIRT3 is a mitochondrially localized NAD+‐dependent deacetylase [16]. SIRT3 substrates include proteins involved in amino acid metabolism (GDH, AceCS2) and electron transport chain (SDH, NDUFA9), as well as the antioxidant proteins, manganese superoxide dismutase 2 (SOD2), and isocitrate dehydrogenase 2 (IDH2) [17, 18, 19, 20, 21, 22, 23], highlighting the role of SIRT3 in maintaining energy homeostasis. Loss of SIRT3, or reduced SIRT3 activity due to lower NAD+/NADH levels, accelerates tumor formation, accompanied by heightened ROS. This effect is attributed to reduced SOD2 activity, a ROS detoxifying enzyme that is deacetylated by SIRT3 [24, 25].

Signal transducer and activator of transcription 3 (STAT3) is a transcription factor that regulates numerous biological functions, including tumorigenicity and inflammation [26]. STAT3 is activated by phosphorylation of tyrosine 705 (Y705) and serine 727 (S727) by protein kinases regulated by cytokine and growth factor receptors and by some oncogenes. Tyrosine‐phosphorylated STAT3 is considered the major transcriptionally active form and can contribute to oncogenesis [27], although nonphosphorylated STAT3 has also been implicated in transcriptional regulation and cancer [28]. STAT3 is constitutively activated in many cancers, including breast cancer [29]. For instance, the IL‐6/STAT3 signaling axis contributes to the growth of stem cell‐like breast cancer growth [30].

In addition to its nuclear functions, a pool of STAT3 localizes to mitochondria [31, 32] where it contributes to a number of cellular functions, including mitochondrial respiration via modulation of complex I, II and V, maintenance of overall mitochondrial health through mPTP closure, and regulation of mitochondrial gene expression, and these functions contribute to Ras‐mediated cellular transformation [33, 34, 35, 36]. Moreover, mitochondrial STAT3 also regulates cellular redox homeostasis. Cells lacking STAT3 display increased ROS levels, both mitochondrial and cellular, and lower levels of GSH [37]. The increased ROS levels can be attributed to a dysfunctional electron transport chain, reduced GSH levels or both, and it remains unclear how mitochondrial STAT3 regulates the cellular GSH pool. While it is known that increased oxidative stress can activate nuclear STAT3 through phosphorylation on Y705, probably through transient inactivation of protein tyrosine phosphatases [38], it remains unclear whether STAT3 contributes to antioxidant gene expression, either through its nuclear or mitochondrial functions. Mechanisms through which metabolic changes in mitochondria are communicated to the nucleus to influence gene expression are complex [39], and it is unclear how mitochondrial STAT3‐regulated process can affect expression of GSH biosynthetic genes, which are nuclear encoded.

In this study, we show that the presence of STAT3 in mitochondria affected the basal expression of the GSH biosynthetic genes *GCLC* and *GCLM,* and of two major antioxidant genes, *NQO1* and *HMOX1*. This regulation was independent of STAT3 nuclear action and did not appear to involve changes in NRF2. We found that compromised complex I activity in the absence of mitochondrial STAT3 affected NAD+ regeneration, resulting in reduced NAD+/NADH ratios, which correlated with impaired antioxidant gene expression and reduced malignant cell survival and migration. Moreover, mitochondrial STAT3 interacted with the dehydrogenase subunit of complex I.

## 2 Materials and methods

### 2.1 Materials

All chemicals were purchased from Sigma‐Aldrich (St. Louis, MO, USA) unless otherwise specified. Reagents with limited water solubility were initially dissolved in dimethylsulfoxide or ethanol before dilution in cell growth media (final solvent concentrations, < 0.1%).

### 2.2 Cell culture

Cells were cultured in Dulbecco's Modified Eagle Medium (DMEM; GE Healthcare, Piscataway, NJ, USA) supplemented with 10% Bovine Calf Serum (Sigma‐Aldrich) and Gentamicin (Cellgro, Corning Life Sciences, Tewksbury, MA, USA) in a 95% air/5% CO_2_ humidified atmosphere. The human triple‐negative breast cancer cell line MDA‐MB‐231 and human lung cancer cell line A549 were obtained from the American Type Culture Collection (Manassas, VA, USA). Cells stably expressing Cas9 and guide RNA against *STAT3* (gRNA sequence: AGATTGCCCGGATTGTGGCC) or a Cas9 alone (siEV) were maintained in the above media containing 5 μg·mL^−1^ of puromycin. MDA‐MB‐231 cells stably expressing STAT3 WT or mitochondrial targeting sequence (MTS) were maintained in the above media containing 200 µg·mL^−1^ of hygromycin. Cells were treated with SIRT1 inhibitor EX‐527 (Cayman Chemicals, Ann Arbor, MI, USA) or SIRT3 inhibitor 3‐TYP (TargetMol), where indicated in the figure legends.

### 2.3 Cell viability assay

Cell viability was measured in triplicate by crystal violet staining, as previously described [40]. In brief, cells seeded in 96‐well plates (Corning Incorporated, Corning, NY, USA) at a density of 10^4^ cells/well were allowed to attach overnight (16 h at 37 °C), followed by addition of 0.375 mm hydrogen peroxide with or without supplementation with N‐Acetyl cysteine (NAC) (25 mm), Trolox (250 µm), or NAM (10 mm), and then cultured for an additional 8 h at 37 °C, followed by staining with 50 µL per well of crystal violet.

### 2.4 Measurements of ROS generation in living cells

Cells were cultured in 6‐well plates for 16 h and then exposed to NAC (25 mm), Trolox (250 µm), or NAM (10 mm) for 8 h. Cells were labeled with 2.5 µm MitoSox Red (Thermo Fisher, Waltham, MA, USA) for 30 min at 37 °C, according to the manufacturer's instructions, and fluorescent intensities were measured by flow cytometry.

### 2.5 Measurement of mitochondrial membrane potential

The mitochondrial membrane potential was measured in triplicate samples using the cationic, cell‐permeant dye tetramethylrhodamine ethyl ester (TMRE) from Thermo Fisher Scientific as previously described [31]. In brief, cells grown in DMEM overnight were washed with PBS, and incubated with 250 nm TMRE for 30 min at 37 °C for dye loading. The cells were washed with PBS prior to being collected for fluorescence analysis by flow cytometry.

### 2.6 Gene expression analyses

For quantitative reverse transcriptase‐polymerase chain reaction (RT‐PCR) analyses, total RNA was isolated using TriZol (Thermo Fisher) and reverse‐transcribed with Moloney murine leukemia virus (M‐MLV). The resulting cDNAs were amplified by qRT‐PCR with SYBR green (Molecular Probes, Eugene, OR, USA) using the primers shown in Table S1. Relative expression was determined by comparison to a standard curve generated from serial dilutions of cDNA containing abundant target sequences, and normalized to the expression of beta‐2‐microglobulin (B2M).

### 2.7 Mitochondrial preparation

Cells were harvested and washed once with PBS. The cell pellet was resuspended in mitochondria purification buffer (MPB: 10 mm Tris, 1 mm EGTA, 200 mm sucrose, pH 7.4) containing freshly added protease inhibitors (Thermo Fisher), 2 mm Na_3_VO_4_, 1 mm DTT, and 1 mm sodium‐β‐glycerophosphate, and incubated on ice for 10 min. The cell suspension was disrupted with 40 strokes in a Dounce homogenizer and centrifuged at 800 ***g*** for 5 min to pellet nuclei and unbroken cells. The supernatant was transferred to a fresh tube and spun at 10 000 ***g*** for 10 min to pellet mitochondria. Mitochondria were resuspended in MPB with 0.02% digitonin and incubated on ice for 5 min, and spun at 10 000 ***g*** for 10 min. Mitochondria were washed 2× with MPB to remove any digitonin, and protein content was quantified by using the Bio‐Rad Coomassie dye assay.

### 2.8 Western Blot analysis

For western blot analysis, mitochondria or cell pellets were lysed in RIPA buffer (50 mm Tris‐HCl, 150 mm NaCl, 0.2% SDS, 0.5% sodium deoxycholate, 1% Triton X‐100, 5% glycerol) containing freshly added protease inhibitors (Thermo Fisher), 2 mm Na_3_VO_4_, 1 mm DTT and 1 mm sodium‐β‐glycerophosphate, and incubated on ice for 10 min. Lysates were spun at 20 000 ***g*** for 10 min, and clarified lysates were resolved on SDS/PAGE, transferred to Polyvinylidene fluoride membranes, blocked with 5% milk, and probed with the antibodies. Unless otherwise specified, all antibodies and detection reagents were used at 1 : 2000 dilution prepared in 5% BSA in TBST: STAT3‐alpha (D1A5) (CST, Danvers, MA, USA, catalog no. 8768S), STAT3 (124H6) (CST, catalog no. 9139S), NDUFV2 (ABclonal, Woburn, MA, USA, catalog no. A7442), NDUFS2 (ABclonal, catalog no. A12858), NDUFAF2 (ABclonal, catalog no. A14296), SDHA (ABclonal, catalog no. A2594), Tubulin (Sigma, catalog no. 26628228), Streptavidin‐HRP (BD, Franklin Lakes, NJ, USA, catalog no. 554066). The blots were developed using a chemiluminescent detection kit (Advansta WesternBright ECL HRP substrate) on a LI‐COR Odyssey® Fc Imaging System.

### 2.9 BioID fusion protein and mass spectrometry analysis

STAT3‐BioID (SB) was constructed by fusing the sequence for MTS‐STAT3 [31] to BioID2 [41] in a lentiviral vector carrying puromycin resistance. MTS‐BioID was constructed by fusing the sequence for MTS [31] to BioID2 [41] in a lentiviral vector carrying puromycin resistance. MDA‐MB‐231 STAT3 WT cells were infected to stably express SB or MTS‐BioID and were maintained under 5 µg·mL^−1^ of puromycin.

MDA‐MB‐231 cells expressing SB or MTS‐BioID were grown in the presence of biotin, and mitochondria were prepared and lysed as described in Section 2.8, except the concentration of SDS was raised to 1%. Biotinylated proteins were collected from mitochondrial lysates on streptavidin Sepharose (Amersham Biosciences, Division of GE Healthcare, Piscataway, NJ, USA), and eluted proteins were identified by mass spectroscopy (MS) as described previously [42, 43].

In Brief, samples were reduced and alkylated with dithiothreitol (1 h at 57 °C) and iodoacetamide (45 min at room temperature). The samples were loaded on a NuPAGE 4–12% Bis‐Tris gel (Thermo Fisher Scientific) and run for 20 min at 200 V. The gel was stained with GelCode Blue Staining Reagent (Thermo Fisher). The gel bands were excised, cut into 1‐mm^3^ pieces, and destained for 15 min in a 1 : 1 (v/v) solution of methanol and 100 mm ammonium bicarbonate. The buffer was exchanged and the samples destained for another 15 min. This was repeated for another three cycles. The gel plugs were dehydrated by washing with acetonitrile and further dried by placing them in a SpeedVac for 20 min.

The samples were digested by adding 250 ng of trypsin (Thermo Fisher) onto the dried gel plugs followed by 300 μL of 100 mm ammonium bicarbonate. Digestion was carried out overnight at room temperature with gentle shaking. The digestion was halted by adding 300 μL of R2 50 μm Poros beads in 5% formic acid and 0.2% trifluoro acetic acid and agitated for 2 h at 4 °C. Beads were loaded onto equilibrated C18 ziptips. The Poros beads were washed with 0.5% acetic acid. The peptides were eluted with 40% acetonitrile in 0.5% acetic acid followed by 80% acetonitrile in 0.5% acetic acid. The organic solvent was removed using a SpeedVac concentrator and the samples reconstituted in 0.5% acetic acid.

An aliquot of each sample was loaded onto an Acclaim PepMap trap column (2 cm × 75 µm) in line with an EASY‐Spray analytical column (50 cm × 75 µm ID PepMap C18, 2 μm bead size) using the auto sampler of an EASY‐nLC 1000 HPLC (Thermo Fisher Scientific) with solvent A consisting of 2% acetonitrile in 0.5% acetic acid and solvent B consisting of 80% acetonitrile in 0.5% acetic acid. The peptides were gradient eluted into a Q Exactive HF‐X Mass Spectrometer (Thermo Fisher Scientific) using the following gradient: 5–35% in 60 min, 35–45% in 10 min, followed by 45–100% in 10 min. The gradient was held at 100% for another 10 min. MS1 spectra were recorded with a resolution of 45 000, an AGC target of 3e6, with a maximum ion time of 45 ms, and a scan range from 400 to 1500 *m/z*. The MS/MS spectra were collected with a resolution of 15 000, an AGC target of 1e5, maximum ion time of 120 ms, one microscan, 2 *m/z* isolation window, a Normalized Collision Energy of 27, and included charge states from +2 to +7.

#### 2.9.1 MS data analysis

All acquired MS2 spectra were searched against the UniProt human database using Sequest within Proteome Discoverer 1.4. The search parameters were as follows: precursor mass tolerance ± 10 p.p.m., fragment mass tolerance ± 0.02 Da, digestion parameters trypsin allowing two missed cleavages, fixed modification of carbamidomethyl on cysteine, variable modification of oxidation on methionine, and variable modification of deamidation on glutamine and asparagine and a 1% peptide and protein FDR searched against a decoy database. The results were filtered to only include proteins identified by at least two unique peptides and likely contaminates were removed [44]. Known mitochondrial proteins were identified by comparison to the human MitoCarta3.0 database [45] and analyzed for pathway enrichment by using Enrichr [46] and by the Molecular Signatures Database v7.2 [47].

### 2.10 Complex I and complex II activity assay

Two microgram of purified mitochondria was incubated in a 96‐well plate with 100 µL of complex I assay buffer (25 mm KPO_4_, 2 mm KCN, 3.5 g·L^−1^ BSA, 60 µm DCIP, 70 µm DCU, 1 µm antimycin A) with or without 10 µm rotenone for 10 min at 37 °C. Five millimolar NADH was added to start the reaction, and absorbance was measured continuously at 600 nm at 30‐s intervals for 10 min at 37 °C. Complex I activity was defined as: 1 U = 1 µm DCIP reduced per min per µg of protein.

Two microgram of purified mitochondria was incubated in a 96‐well plate with 100 µL of Complex II assay buffer (80 mm KPO_4_, 1 g·L^−1^ BSA, 2 mm EDTA, 0.2 mm ATP, 80 µm DCIP, 50 µm DCU, 1 µm Antimycin A, 3 µm rotenone) for 10 min at 37 °C. Ten millimolar sodium succinate and 0.3 mm KCN were added to start the reaction, and absorbance was measured continuously at 600 nm at 30 s intervals for 10 min at 37 °C. Complex II activity was defined as: 1 U = 1 µm DCIP reduced per min per µg of protein.

### 2.11 Clonogenic assay

Cells grown in DMEM were plated in 6‐well dishes at 1000 cells/well. Colonies were allowed to grow for 10–14 days, with media change every 2 days. After large clones were visible, the colonies were washed with PBS and stained with crystal violet before being counted using ImageJ to determine colony number and size.

### 2.12 Wound healing scratch assay

Cells were plated at 10^5^ cells per well in a 12‐well dish. Following attachment, the cell monolayer was scratched in a straight line using a 200‐µL pipette tip. Cell monolayers were imaged with a ZOE fluorescent cell imager immediately and after 24 h. Images were analyzed using imagej (NIH, Bethesda, MD, USA) to quantify wound closure.

### 2.13 NAD/NADH measurement

Cells were harvested, and NAD+/NADH content was measured by using the NAD/NADH‐Glo Assay kit (Promega, Madison, WI, USA), according to instructions.

### 2.14 GSH measurement

Cells were harvested and GSH content was measured by using the GSH Assay kit (Promega), according to instructions.

### 2.15 NRF2 luciferase reporter gene assay

MDA‐MB‐231 *STAT3* WT and knockout (KO) cells were transfected with a luciferase reporter plasmid programmed by the antioxidant‐responsive element (ARE) sequence from the promoter of the human NAD(P)H quinone oxidoreductase gene, as previously described [48]. In brief, cells were transfected with the ARE‐luciferase plasmid and a TK‐renilla luciferase control plasmid using Lipofectamine 2000. Forty‐eight hours after transfection, both firefly and renilla luciferase activities were measured with the dual luciferase reporter assay kit (Promega), and expression data were calculated from the firefly/renilla luciferase ratios and reported as relative light units (RLU).

### 2.16 Statistical analysis

Experiments were performed in triplicate, and data were presented as the mean ± SD (*n* = 3) and analyzed by unpaired Student’s *t*‐test or one‐way analysis of variance (ANOVA). All data shown are representative of at least three independent biological replicates and were analyzed by using graphpad prism version 8.0 (GraphPad Software, San Diego CA, USA).

## 3 Results

### 3.1 Mitochondrial STAT3 positively regulates complex I function and cellular NAD+ concentration

To determine whether STAT3 is critical for mitochondrial functions in triple‐negative breast cancer (TNBC), we created *STAT3* KO cell lines by CRISPR/Cas9 gene targeting in the TNBC cell line MDA‐MB‐231 [40] and compared them to wild‐type (WT) counterparts. We also generated *STAT3* KO cell lines reconstituted with either WT *STAT3* or with artificially mitochondrially restricted forms of *STAT3* (MTS), including a version that encoded the phosphorylation‐deficient S727A mutation, previously shown to impair mitochondrial STAT3 functions [31]. STAT3 depletion and reconstitution were validated by western blot (Fig. S1A). STAT3 was expressed in reconstituted lines at ~ 25–50% of endogenous levels, with the WT protein expressed in both cytosolic and mitochondrial fractions, while MTS‐STAT3 accumulated exclusively in mitochondria.

**Fig. 1:**
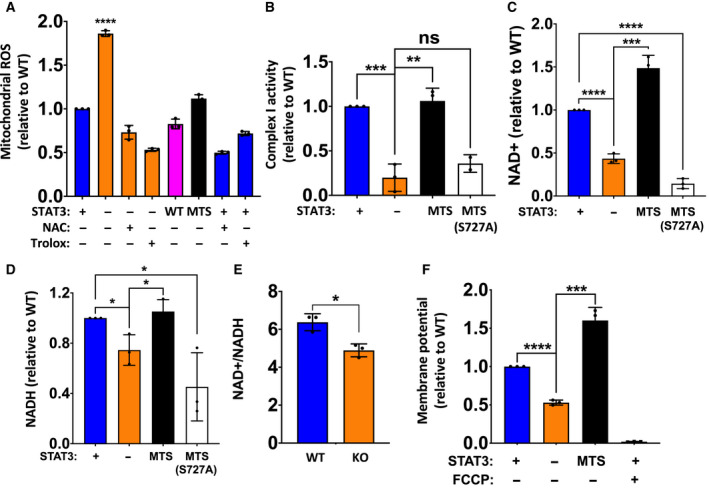
Mitochondrial STAT3 positively regulates complex I function and cellular NAD+ concentration. (A) Mitochondrial ROS measured for *STAT3* WT, KO, KO + WT, and KO + MTS MDA‐MB‐231 cells. *STAT3* WT and KO cells were treated with antioxidants (25 mm NAC or 250 μm Trolox) for 8 h where indicated before measurement. Only *STAT3* KO was significantly different from others (*P* < 0.001, one‐way ANOVA). (B) Complex I activity for *STAT3* WT, KO, KO + MTS (WT), and KO + MTS (S727A) cells. (C) NAD+ and (D) NADH levels in *STAT3* WT, KO, KO + MTS (WT), and KO + MTS (S727A) cells. (E) NAD+/NADH ratio in MDA‐MB‐231 *STAT3* WT and KO cells. (F) Mitochondrial membrane potential measured for *STAT3* WT, KO, and KO + MTS cells using TMRE. Control cells were treated with 50 nm FCCP as indicated for 10 min before measurement. Student's *t*‐test: ****P < 0.0001, ***P < 0.001, **P < 0.01, *P < 0.05, ns = not significant, *n* = 3. Error bars represent ± SD.

Mitochondria are the major site for ROS production in the cell, and impaired electron transport chain activity generates increased ROS [49]. It has also been shown that loss of STAT3 leads to decreased oxidative phosphorylation and increased mitochondrial ROS (mROS) [37]. Therefore, we checked whether the absence of STAT3 induced oxidative stress in MDA‐MB‐231 cells. Cells lacking STAT3 had almost two times more mROS than WT cells (Fig. 1A). This increased mROS accumulation could be prevented by reconstitution with either WT or MTS‐STAT3, and could be quenched by treatment with the antioxidants NAC and Trolox. Since MTS‐STAT3 expression was sufficient to return mROS levels to WT levels, we concluded that mitochondrial STAT3 functions are critical for the regulation of ROS in TNBC.

Complex I is one of the main contributors to mitochondrial superoxide production [50], and has been shown to have reduced activity in the absence of STAT3 [31, 32]. Hence, we measured complex I activity in MDA‐MB‐231 *STAT3* WT and KO cells. Complex I activity was significantly lower in *STAT3* KO cells, reduced to ~ 20% the level in WT cells (Fig. 1B); however, there was no significant change in complex II activity in the absence of STAT3 (Fig. S1B). Importantly, reconstitution of *STAT3* KO cells with MTS‐STAT3 fully restored complex I activity (Fig. 1B), indicating that enzyme activity was dependent on the mitochondrial pool of STAT3 protein. We also tested *STAT3* KO cells reconstituted with the MTS‐STAT3‐S727A mutant, because phosphorylation of this residue has been implicated in mitochondrial STAT3 functions [31]. Reconstitution with MTS‐STAT3‐S727A did not restore complex I function (Fig. 1B), confirming a critical role for this residue and probably its phosphorylation in the regulation of complex I activity by STAT3 in TNBC. Although these results documented positive regulation of complex I activity by mitochondrial STAT3, no differences were observed in complex I protein levels (NDUFS2, NDUFV2, NDUFAF2) between *STAT3* WT and KO mitochondria (Fig. S1C). This result suggests that mitochondrial STAT3 enhances complex I enzymatic activity, rather than its synthesis or assembly.

Mitochondrial complex I, through its NADH: quinone oxidoreductase activity, maintains the cellular pool of NAD by oxidizing NADH to NAD+, while coupling the translocation of protons across the mitochondrial membrane [51]. To determine how loss of STAT3 affected cellular dinucleotide pools, we measured NAD+ and NADH levels in *STAT3* WT and KO cells. In the absence of STAT3, dinucleotide levels were reduced, with NAD+ levels reduced to ~ 40% of WT levels (Fig. 1C), while NADH levels were less affected (Fig. 1D). Reconstitution of KO cells with MTS‐STAT3 but not with MTS‐STAT3‐S727A restored NAD+ and NADH concentrations to endogenous levels (Fig. 1C,D). These results document a role for mitochondrial STAT3 in the maintenance of NAD abundance in TNBC cells, possibly through modulation of complex I activity.

Increased mROS due to inhibition of electron transport chain complexes leads to mitochondrial membrane depolarization [52]. Indeed, measurement of mitochondrial membrane potential revealed that *STAT3* KO cells have about 50% lower membrane potential than WT cells (Fig. 1F). Normal membrane potential was restored in *STAT3* KO cells by reconstitution with MTS‐STAT3. Specificity of the membrane potential measurements was confirmed by the reduced potential observed following treatment with the ETC decoupling agent, FCCP (Fig. 1F).

### 3.2 Mitochondrial STAT3 regulates expression of GSH biosynthetic and antioxidant genes

NAD+ abundance is critical for expression of antioxidant genes, including genes belonging to the GSH biosynthetic pathway [14]. Therefore, we investigated whether the observed dinucleotide reductions in MDA‐MB‐231 *STAT3* KO cells impacted the expression of redox genes. To that end, we quantified basal mRNA levels of *GCLC* (glutamate‐cysteine ligase catalytic subunit), *GCLM* (glutamate‐cysteine ligase modifier subunit), *HMOX1* (heme oxygenase 1), and *NQO1* (NAD(P)H quinone dehydrogenase) in *STAT3* WT and KO cells.

**Fig. 2:**
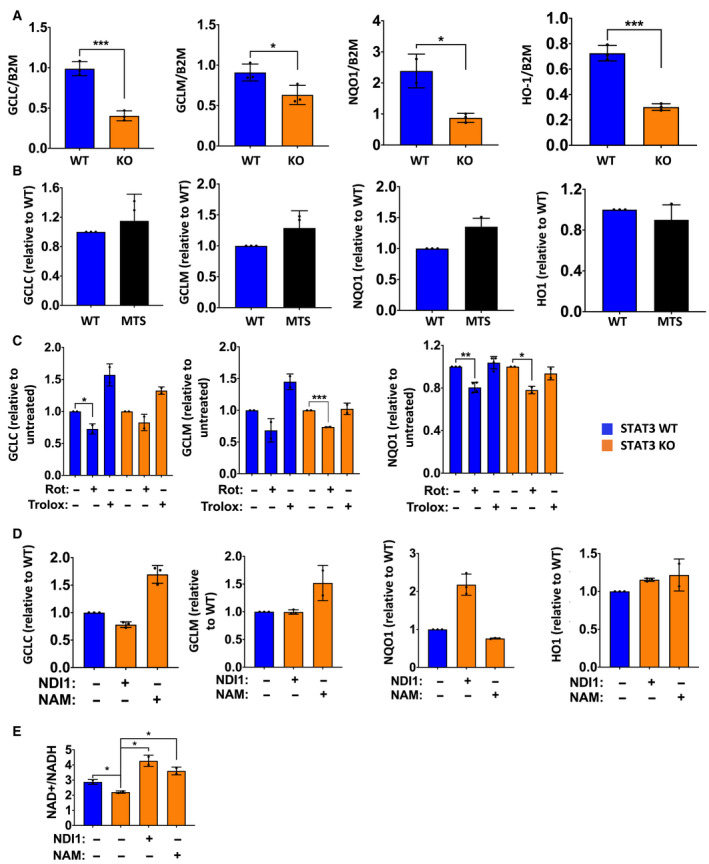
Mitochondrial STAT3 regulates expression of GSH biosynthetic genes and antioxidant genes. (A) Basal mRNA levels of GSH biosynthesis pathway genes (*GCLC, GCLM, NQO1*, and *HMOX1*) in MDA‐MB‐231 *STAT3* WT and KO cells. (B) mRNA levels of antioxidant genes in MDA‐MB‐231 *STAT3* KO cells after reconstitution with MTS‐STAT3. (C) mRNA levels of antioxidant genes in MDA‐MB‐231 *STAT3* WT and KO cells after treatment with 50 µm rotenone or 250 µm Trolox for 8 h. (D) mRNA levels of antioxidant genes in MDA‐MB‐231 cells after reconstitution with NDI1 or treatment with 10 mm nicotinamide (NAM) for 16 h. (E) NAD+/NADH ratio measured in *STAT3* WT, KO, KO + NDI1, or KO cells treated with 10 mm NAM for 48 h. Student’s *t*‐test: ***P < 0.001, **P < 0.01, *P < 0.05, ns = not significant, *n* = 3. Error bars represent ± SD.

Given the heightened oxidative stress observed in *STAT3* KO cells (Fig. 1), we anticipated an induction of antioxidant gene expression, as increased ROS burden can induce transcriptional activation of genes involved in antioxidant synthesis through activation of NRF2 [53]. In contrast, we observed a significant reduction (50–80%) in the expression of these genes in cells lacking STAT3 (Fig. 2A). We also measured cellular GSH and found that MDA‐MB‐231 *STAT3* KO cells had ~ 50% lower amounts of cellular GSH (Fig. S2A), as observed previously in other cancer cells [37]. To confirm the direct involvement of mitochondrial STAT3 in gene regulation, antioxidant gene expression was measured in KO cells reconstituted with MTS‐STAT3. Expression of all four genes was restored to WT levels following expression of MTS‐STAT3 (Fig. 2B). Consistent with the restored mRNA levels, GSH concentration was also restored by reconstitution with MTS‐STAT3 (Fig. S2A). We confirmed the inability of MTS‐STAT3 to restore expression of conventional STAT3 target genes, when reconstituted in *STAT3* KO cells. Loss of STAT3 led to a significant decrease in *SOCS3* mRNA levels, which were restored by WT‐STAT3 but not by MTS‐STAT3 (Fig. S2B). This result demonstrated that mitochondrial STAT3 impacts the regulation of these nuclear‐encoded genes, in spite of its lack of direct nuclear transcriptional function [54].

We wanted to understand whether the loss of antioxidant gene expression could be attributed to the reduced complex I activity observed in STAT3 KO cells. To that end, we measured the mRNA levels of these genes in *STAT3* WT and KO cells treated with the complex I inhibitor, rotenone. Treatment of cells with 50 µm rotenone for 8 h caused a modest reduction (~ 20–30%) in the expression of these genes (Fig. 2C) that mirrored the reductions observed in the absence of STAT3. We also treated both *STAT3* WT and KO cells with the cell‐permeable vitamin E analog and free radical scavenger, Trolox, to determine whether mitigating the increased ROS in *STAT3* KO cells could rescue gene expression. Treating cells with 250 µm Trolox for 8 h caused a modest increase in the expression of *GCLC* in both *STAT3* WT and KO cells, but it did not restore its level in KO cells to those seen in WT cells (Fig. 2A). Moreover, Trolox treatment had no impact on *GCLM* or *NQO1* in either WT or KO cells (Fig. 2C).

Since ROS levels did not appear to be responsible for changes in antioxidant gene expression, we tested whether reduced levels of NAD+ were responsible for impaired gene expression. To check this, we augmented NADH dehydrogenase activity in *STAT3* KO cells by ectopically expressing yeast *NDI1*. Yeast NDI1 is a rotenone‐resistant and STAT3‐independent NADH dehydrogenase that is capable of bypassing defects in complex I function when expressed in mammalian cells [40, 55, 56]. Interestingly, NDI1 expression restored antioxidant gene expression in *STAT3* KO cells to WT levels (Fig. 2D). We also treated *STAT3* KO cells with nicotinamide (NAM), a precursor capable of boosting cellular NAD+ levels [57]. Treatment of *STAT3* KO cells with 10 mm NAM restored expression of antioxidant genes to WT levels (Fig. 2D). Treatment with NAM or expression of NDI1 in KO cells also normalized NAD+/NADH ratios (Fig. 2E).

### 3.3 STAT3‐dependent antioxidant gene expression is not mediated by changes in NRF2 function

**Fig. 3:**
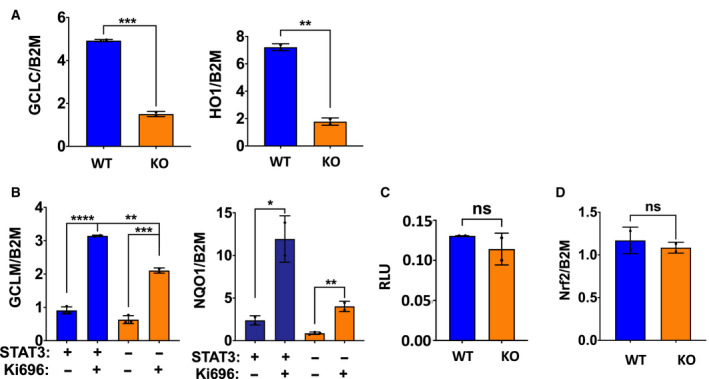
STAT3‐dependent antioxidant gene expression is not mediated by changes in NRF2 function. (A) Basal mRNA expression of antioxidant genes in NSCLC cell line A549 *STAT3* WT and KO. (B) mRNA expression of antioxidant genes in MDA‐MB‐231 *STAT3* WT and KO cells treated with 1 μm of the NRF2 activator, KI696 for 48 h. (C) Luminescence assay in MDA‐MB‐231 *STAT3* WT and KO cells using a reporter plasmid responsive to NRF2. Luciferase data are expressed as RLU. (D) Basal mRNA levels of *NRF2* in MDA‐MB‐231 STAT3 WT and KO cells. Student’s *t*‐test: ****P < 0.0001, ***P < 0.001, **P < 0.01, *P < 0.05, ns = not significant, *n* = 3. Error bars represent ± SD.

Since the antioxidant genes observed to have lower expression in the absence of STAT3 are regulated by NRF2 during oxidative stress, we determined the status of the NRF2 pathway in cells lacking STAT3. First, we examined antioxidant gene expression in the non‐small‐cell lung carcinoma cell line, A549, which displays constitutive NRF2 activity due to a null mutation in the negative regulator, *KEAP1* [58]. Similar to MDA‐MB‐431 cells, A549 cells expressed reduced levels of antioxidant genes *GCLC* and *HO1* when the *STAT3* gene was disrupted (Fig. 3A), in spite of the presence of constitutively active NRF2.

We also confirmed that the NRF2 pathway is not rendered defective by the absence of STAT3 by treating cells with a small molecule activator of NRF2, KI696 [59]. KI696 treatment induced robust expression of antioxidant genes in both WT and KO cells (Fig. 3B), consistent with the NRF2 pathway remaining functional in these cells regardless of the status of STAT3. However, while fold induction of *GCLM* and *NQO1* in response to KI696 treatment was equivalent between WT and KO cells, their absolute levels were lower in KO cells reflecting impaired basal gene expression in the absence of STAT3.

We also measured the basal activity of NRF2 using a reporter assay. MDA‐MB‐231 *STAT3* WT and KO cells were transfected with a luciferase reporter driven by NRF2‐responsive promoter elements [48]. Both WT and KO cells transfected with this reporter expressed similar levels of luciferase (Fig. 3C), demonstrating that basal NRF2 transcriptional activity was equivalent in the two cell lines. Finally, measurement of *NRF2* mRNA levels in *STAT3* WT and KO cells demonstrated no differences in expression between the two cell lines (Fig. 3D). Overall, these results exclude an impairment in the NRF2 pathway as a primary cause for reduced basal antioxidant gene expression in cells lacking STAT3 and indicate a requirement for STAT3 in addition to NRF2.

### 3.4 Mitochondrial STAT3 regulates clonogenic potential and sensitivity to oxidative stress

**Fig. 4:**
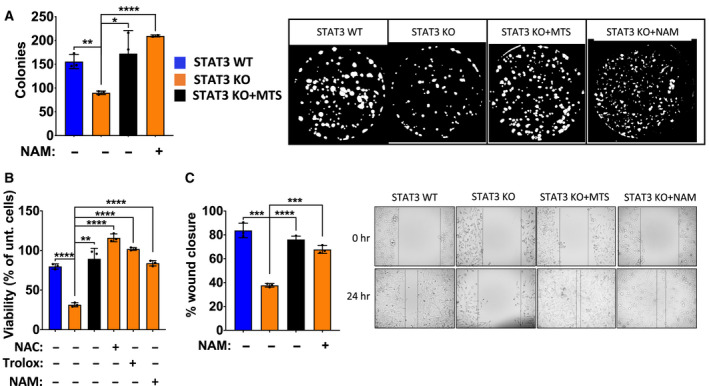
Mitochondrial STAT3 regulates clonogenic potential and sensitivity to oxidative stress. (A) Number of colonies formed by MDA‐MB‐231 *STAT3* WT, KO, KO + MTS cells, and KO cells treated with 10 mm NAM for 10 d. Representative image from ImageJ is shown. (B) Cell viability measured by crystal violet of MDA‐MB‐231 *STAT3* WT, KO, and KO + MTS cells treated with 0.375 mm H_2_O_2_ or 0.375 mm H_2_O_2_ + antioxidants (25 mm NAC, 250 μm Trolox, or 10 mm NAM) for 8 h. (C) Wound healing scratch assay measured for MDA‐MB‐231 *STAT3* WT, KO, KO + MTS cells, and KO cells treated with 10 mm NAM for 24 h. Student’s *t*‐test: ****P < 0.0001, ***P < 0.001, **P < 0.01, *P < 0.05, ns = not significant, *n* = 3. Error bars represent ± SD.

To explore the biological consequences of mitochondrial STAT3 function, we examined its requirement for cell growth and survival. To examine clonogenic potential, we plated WT and KO cells at limiting dilution and measured colony formation after 10 days. *STAT3* KO cells showed reduced clonogenic potential, resulting in the growth of 50% fewer colonies (Fig. 4A). Importantly, WT levels of clonogenic growth were restored by expression of MTS‐STAT3. Moreover, the diminished clonal growth of *STAT3* KO cells was also reverted by culturing the cells in the presence of NAM. Nonetheless, there were no significance differences in the proliferation rates of *STAT3* WT and KO cells when cultured under standard growth conditions (Fig. S2C). We also found that *STAT3* KO cells undergo 50% more cell death following oxidative stress, by exposing cells to H_2_O_2_ (Fig. 4B). Cell viability could be restored by countering the oxidative stress with antioxidants (25 mm NAC or 250 µm Trolox; Fig. 4B), consistent with the enhanced sensitivity to stress being due to the altered redox balance in *STAT3* KO cells. We also treated *STAT3* KO cells with NAM prior to exposure to the oxidative stress. Consistent with expectations, NAM pretreatment also protected *STAT3* KO cells from peroxide‐induced cell death (Fig. 4B). Finally, we examined whether loss of STAT3 reduced cellular migration by performing a wound healing assay [60]. *STAT3* KO cells migrated twofold less that WT cells, when migration potential was measured after 16 h (Fig. 4C). Similar to the role of mitochondrial STAT3 and NAD in clonal growth, the reduced migration of *STAT3* KO cells was restored either by expression of MTS‐STAT3 or by supplementation with NAM. Together, these results show that mitochondrial STAT3, through its ability to maintain normal pools of NAD+, regulates clonal growth, oxidant resistance, and cell survival and migration.

### 3.5 Mitochondrial STAT3 interacts with complex I components

**Fig. 5:**
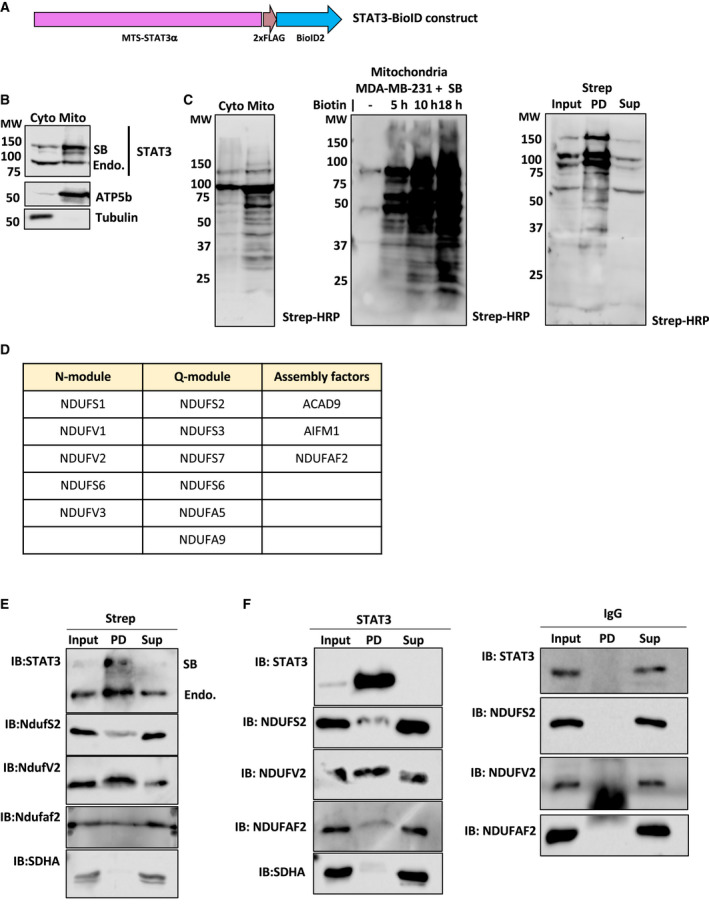
Mitochondrial STAT3 interacts with complex I components. (A) Cartoon of the STAT3‐BioID construct (SB). (B) Western blot showing cytosolic and mitochondrial fractions from MDA‐MB‐231 WT cells expressing SB. The SB construct is larger in size than the endogenous protein (indicated in the figure) and is mainly located in the mitochondrial fraction. (C) Streptavidin‐HRP blot for cells expressing SB and treated with 50 μm biotin for 18 h showing that biotinylated proteins are mainly restricted to the mitochondrial fraction (left). Time course of biotin treatment showing time‐dependent increase in biotinylated proteins (middle). Pull‐down with streptavidin sepharose from mitochondrial lysates expressing SB and treated with 50 μm biotin for 18 h showing near quantitative recovery of biotinylated proteins (right). (D) Table showing list of complex I proteins biotinylated in the presence of SB. (E) Streptavidin sepharose was used to pull‐down biotinylated proteins from mitochondrial lysates of MDA‐MB‐231 *STAT3* WT cells expressing SB treated with 50 μm biotin for 18 h, and complex I components (NDUFV2, NDUFS2, NDUFAF2) were identified by immunoblotting. (F) Left: Immunoprecipitation of endogenous STAT3 verifies interaction with complex I components (NDUFV2, NDUFS2, NDUFAF2). Right: Pull‐down with IgG as control. Mw are expressed in KDa.

To explore how mitochondrial STAT3 could regulate complex I activity in TNBC, we examined protein partners of MTS‐STAT3. To this end, we fused MTS‐STAT3 to the BioID2 protein to create a proximity‐labeling probe, STAT3‐BioID (SB) (Fig. 5A), and expressed it in MDA‐MB‐231 cells. The fusion protein was confirmed to localize to mitochondria (Fig. 5B), and biotinylation of target proteins was mainly observed in the mitochondrial fraction (Fig. 5C). Total protein biotinylation increased in response to treatment time with biotin (Fig. 5C), and biotinylated proteins could be quantitatively recovered from mitochondrial extracts by streptavidin pull‐down (Fig. 5C).

To evaluate proteins in the vicinity of STAT3, SB‐expressing cells were treated with biotin, and the biotinylated proteins recovered by streptavidin pull‐down were identified by MS (Data S1). We used the Enrichr and Molecular Signatures enrichment analysis tools [46, 47, 61] to identify pathways defined by the proteins preferentially biotinylated by mitochondrial STAT3. Proteins associated with oxidative phosphorylation were detected, due in part to enrichment of a number of complex I subunit components. Of the 17 protein subunits of the oxidoreductase (N/Q) module of complex I involved in NADH oxidation and ubiquinone reduction [62], 11 were present in the biotinylated fraction (Fig. 5D).

To confirm these interactions, biotinylated proteins from mitochondrial lysates of cells treated with 50 µm biotin for 18 h were recovered on streptavidin beads and identified by western blotting. NDUFS2, NDUFV2, and NDUFAF2, three constituents or assembly factors of complex I identified by mass spectrometry, were independently confirmed to be proximity labeled by STAT3‐BioID (Fig. 5E). Interestingly, endogenous STAT3 was detected in the biotinylated fraction, indicating that the SB fusion protein interacted with endogenous STAT3, possibly due to dimerization of mitochondrial STAT3, as previously suggested [35]. In contrast, the complex II component SDHA was not recovered in the pull‐down fraction (Fig. 5E). As control, we used mitochondrial lysates from cells expressing a MTS‐BioID construct lacking STAT3. Mitochondrial lysates from these cells were biotinylated (Fig. S3, right panel), but pull‐down using streptavidin beads did not recover any complex I proteins or STAT3 (Fig. S3, left panel). To confirm these interactions with endogenous STAT3, mitochondrial lysates from MDA‐MB‐231 cells were immunoprecipitated using an antibody against STAT3. NDUFS2, NDUFV2, and NDUFAF2 were all co‐immunoprecipitated with STAT3, while SDHA was not (Fig. 5F). None of these proteins were recovered in IgG control immunoprecipitation experiments (Fig. 5F, right panel). Mitochondrial STAT3 interacted most strongly with NDUFV2, whereas the interaction with NDUFS2 and the complex I assembly factor NDUFAF2 appeared weaker (Fig. 5F). Notably, although STAT3 was quantitatively recovered by immunoprecipitation, a significant fraction of these endogenous complex I proteins were left in the supernatant. Overall, these results indicate that mitochondrial STAT3 interacts nonstoichiometrically with a portion of complex I. Interestingly, the complex I components identified as STAT3 interactors are associated with the dehydrogenase module, consistent with altered dehydrogenase activity in the absence of STAT3.

## 4 Discussion

It has become increasingly clear that cancer cells display increased ROS production and that elevated ROS contributes to malignancy by augmenting many of the characteristics of cancer cell behavior [1, 2]. However, ROS are also harmful, so an increased redox potential is essential for cancer cell survival [63]. One of the major cellular sources of ROS is mitochondrial metabolism, due to ROS generation through the activity of the respiratory chain, largely due to complexes I and III [49]. Several studies have demonstrated increased ROS in tumor cells following loss of STAT3, and this effect has been shown to be mediated, at least in part, by mitochondrial STAT3 [64], which regulates the activity of complex I [31, 32]. Inefficient complex I function due to the absence of STAT3 can therefore lead to increased mitochondrial ROS, and mitochondrial STAT3 has been proposed to act as a redox sensor [65, 66]. Another source of increased ROS in the absence of STAT3 are decreased GSH levels, due to reduced ROS buffering capacity [37]. However, the basis for the decreased GSH observed in the absence of STAT3 has remained unknown.

In this study, we found that mitochondrial STAT3 modulates GSH levels at least in part by regulating the expression of genes involved in GSH synthesis. However, STAT3 did not act directly as a transcription factor, since mitochondrial STAT3 was sufficient to maintain expression of these nuclear genes. Instead, regulation of gene expression must depend on a retrograde signal that facilitates communication between mitochondria and nuclei [39], a process that promotes adaptation to metabolic flux. Retrograde signaling can be direct, in which a mitochondrially localized transcription factor is sent to the nucleus under appropriate circumstances [67]. Retrograde signaling can also occur through a less direct route, in which a mitochondrial metabolite influences cellular responses, including changes in nuclear gene expression [68, 69]. Our data suggest that NAD+ serves this function to regulate GSH synthesis. STAT3 controlled the abundance of cellular NAD+ through maintenance of complex I dehydrogenase activity. Absence of STAT3 led to reduced complex I activity, resulting in impaired regeneration of NAD+ from NADH oxidation, leading to a reduced overall NAD+/NADH ratio. Importantly, normal NAD+/NADH ratios could be restored by expression of mitochondrially localized STAT3, increased NAD synthesis due to nutritional supplementation with the NAD precursor NAM, or by complementing impaired complex I function through expression of the STAT3‐independent NADH dehydrogenase NDI1 from yeast. All of these approaches to restoring a WT NAD+/NADH ratio in *STAT3*‐null cells led to normalized antioxidant gene expression.

NAD+ has been shown previously to modulate nuclear gene expression through a number of mechanisms. The sirtuin (SIRT) family of histone deacetylases require NAD+ as a cofactor for enzymatic activity, thereby linking epigenetic regulation to the abundance of NAD+ [13, 70]. Similarly, C‐terminal binding protein (CtBP) is a NAD+‐regulated transcriptional corepressor [71] that links transcriptional activity to the cellular NAD+/NADH ratio. NAD has also been shown to regulate DNA methylation, with lower levels of NAD correlating with increased methylation and therefore reduced gene transcription [72]. SIRT can also regulate gene expression by deacetylating transcription factors rather than histones, such as FoxO3 and peroxisome proliferator‐activated receptor‐gamma co‐activator‐1alpha (PGC1α) [73]. Sirt3 has been shown to reduce oxidative damage through IDH2 [21]. Sirt3 increases IDH2 activity through deacetylation, thereby increasing mitochondrial NADPH levels and GSSG/GSH ratio. However, the mitochondrial STAT3‐dependent expression of GCLC was minimally affected by inhibition of SIRT1 or SIRT3 (Fig. S4A), although inhibition of these enzymes decreased the expression of a typical SIRT target gene, *PGC1α* (Fig. S4B) [74, 75, 76].

Our data suggest that the NAD+/NADH ratio modulated by mitochondrial STAT3 through changes in complex I activity regulates antioxidant gene expression. Indeed, NAD‐dependent regulation of antioxidant gene expression has been previously reported [13, 77, 78], and was ascribed to changes in the abundance of the NRF2 transcription factor. However, we did not observe an impairment of NRF2 levels nor changes in NRF2 transcriptional activity in STAT3 KO cells (Fig. 3B,C), suggesting that STAT3 acts on other components of this pathway and not by directly modulating NRF2.

Mitochondrial STAT3 regulates the electron transport chain, including complex I activity [31, 32], but the mechanistic basis for this regulation remains unclear. Our results show that complex I activity and antioxidant gene expression depend on mitochondrial STAT3 Ser727 phosphorylation, previously shown to be critical for supporting malignant transformation [31, 79]. Moreover, our proximity‐labeling studies revealed that mitochondrial STAT3 associates with three subunits of the NADH dehydrogenase arm of complex I, and this interaction was confirmed by examining endogenous proteins. Importantly, we did not observe any changes in the abundance of complex I subunits between *STAT3* WT and KO cells, suggesting that STAT3 influences complex I function and not assembly in these cells. Given the low abundance of STAT3 relative to complex I, this interaction is not stoichiometrically equivalent [80], and the majority of complex I subunit proteins were not associated with STAT3. It is possible that STAT3 associates with a subset of complex I that is critical for dehydrogenase activity. For instance, it has been suggested that STAT3 associates with respiratory supercomplexes that are important for reducing electron leak and thereby limiting ROS generation [81].

TNBC is the most aggressive form of breast cancer, lacking the expression of estrogen receptor, progesterone receptor, and human epidermal growth factor receptor 2. STAT3 has been shown to play an important pro‐oncogenic role in TNBC [82] and to be a potential therapeutic target [83], with studies indicating the presence of hyperactivated STAT3, driven by the phosphorylation of tyrosine 705. In addition, S727‐phosphorylated mitochondrial STAT3 has also been implicated in breast cancer [84].

We observed a significant requirement for mitochondrial STAT3 in the maintenance of clonogenecity and wound healing in MDA‐MB‐231, confirming that the mitochondrial fraction of STAT3 plays an important role in driving tumorigenecity in TNBC. Importantly, the requirement for mitochondrial STAT3 in these processes could be circumvented by modulation of NAD+ levels through supplementation with NAM or restoration of dehydrogenase activity. These results could have therapeutic implications, as it has been shown that targeting NAD+ metabolism can sensitize tumor cells to drug and radiation therapy [85, 86], adding another potential rationale for targeting mitochondrial STAT3 during cancer therapy [40].

## 5 Conclusions

Overall, our study presents a novel role for mitochondrial STAT3 in regulation of cellular redox balance through modulation of antioxidant gene expression as a consequence of complex I activity. Reduced complex I activity in the absence of mitochondrial STAT3 impaired regeneration of NAD+. Perturbed NAD+/NADH ratios failed to maintain the basal expression of antioxidant genes, leading to a diminished cellular reducing capacity, a more oxidized cellular environment, increased vulnerability to oxidative stress, and reduced cellular attributes of malignancy. These results implicate NAD+ as a retrograde signal relaying the status of mitochondrial metabolism to the nucleus, and they highlight the potential of mitochondrial STAT3 as a target for cancer chemotherapy.

## Conflict of interest

The authors declare no conflict of interest.

## Author contributions

TL and DEL conceived and designed the project. TL, JA, and BU acquired the data. TL, JA, BU, MA, LB, and DEL analyzed and interpreted the data; and TL, LB, and DEL wrote the paper.

### Peer Review

The peer review history for this article is available at https://publons.com/publon/10.1002/1878‐0261.12928.

## Supporting information


**Fig. S1.** Characterization of mutant and reconstituted cell lines. (A) Western blot to confirm MDA‐MB‐231 *STAT3* KO and reconstitution of KO cells with WT, MTS (WT), or MTS (S727A) STAT3 (*left panel:* cytosolic fraction; *right panel:* mitochondrial fraction). Mitochondrial STAT3 levels are normalized to SDHA. (B) Complex II activity for *STAT3* WT, KO, KO + MTS (WT), and KO + MTS (S727A) cells. (C) Western blot to confirm expression levels of complex I subunits in MDA‐MB‐231 *STAT3* WT and KO cells. Student’s *t*‐test: ****= *P* < 0.0001, ***= *P* < 0.001, **= *P* < 0.01, *= *P* < 0.05, ns = not significant, *n* = 3. MW are expressed in KDa. Error bars represent ± SD.
**Fig. S2.** (A) Glutathione levels in WT and mutant cell lines. GSH levels measured in *STAT3* WT, KO and KO + MTS cells. (B) Growth of cells is unaffected by STAT3. Growth curve for MDA‐MB‐231 *STAT3* WT and KO cells over 4 d measured using crystal violet. (C) Induction of *SOCS3* gene expression depends on nuclear STAT3. mRNA expression of nuclear STAT3 target gene *SOCS3* in MDA‐MB‐231 *STAT3* WT, KO, KO + WT, and KO + MTS cells. Student’s *t*‐test: ****= *P* < 0.0001, ***= *P* < 0.001, **= *P* < 0.01, *= *P* < 0.05, ns = not significant, *n* = 3. Error bars represent ± SD.
**Fig. S3.** Antioxidant gene expression does not require SIRT1 or SIRT3 activity. (A) mRNA expression of *GCLC* in MDA‐MB‐231 *STAT3* WT or KO cells treated with 200 μm EX‐527 (SIRT1 inhibitor) or 200 μm 3‐TYP (SIRT3 inhibitor) for 24 h. (B) mRNA expression of *PGC1*α in MDA‐MB‐231 *STAT3* WT cells treated with 200 μm EX‐527 or 200 μm 3‐TYP for 24 h. Student’s *t*‐test: ****= *P* < 0.0001, ***= *P* < 0.001, **= *P* < 0.01, *= *P* < 0.05, ns = not significant, *n* = 3. Error bars represent ± SD.
**Fig. S4.** Specificity control for STAT3‐BioID pull‐down. Streptavidin sepharose was used to pull‐down biotinylated proteins from mitochondrial lysates of MDA‐MB‐231 *STAT3* WT cells expressing MTS‐BioID treated with 50 μm biotin for 18 h, and complex I components (NDUFV2, NDUFS2, NDUFAF2) were identified by immunoblotting (*left*). Pull‐down with streptavidin sepharose from mitochondrial lysates expressing MTS‐BioID and treated with 50 μm biotin for 18 h showing near quantitative recovery of biotinylated proteins (*right*).
**Table S1.** Primer sequences for RNA quantification by qRT‐PCR.

**Data S1.** MS/MS data for peptides copurifying with STAT3‐BioID.
