# Supplementary material for: Mitochondrial STAT3 regulates antioxidant gene expression through complex I‐derived NAD in triple negative breast cancer
Source: Mol Oncol. 2021 Apr 10;15(5):1432–49. doi: 10.1002/1878-0261.12928 (PMC8096790; doi:10.1002/1878-0261.12928)
Supplement: Supplementary file 1 — Fig. S1. Characterization of mutant and reconstituted cell lines. (A) Western blot to confirm MDA‐MB‐231 STAT3 KO and reconstitution of KO cells with WT, MTS (WT), or MTS (S727A) STAT3 (left panel: cytosolic fraction; right panel: mitochondrial fraction). Mitochondrial STAT3 levels are normalized to SDHA. (B) Complex II activity for STAT3 WT, KO, KO + MTS (WT), and KO + MTS (S727A) cells. (C) Western blot to confirm expression levels of complex I subunits in MDA‐MB‐231 STAT3 WT and KO cells. Student’s t‐test: ****= P < 0.0001, ***= P < 0.001, **= P < 0.01, *= P < 0.05, ns = not significant, n = 3. MW are expressed in KDa. Error bars represent ± SD. Fig. S2. (A) Glutathione levels in WT and mutant cell lines. GSH levels measured in STAT3 WT, KO and KO + MTS cells. (B) Growth of cells is unaffected by STAT3. Growth curve for MDA‐MB‐231 STAT3 WT and KO cells over 4 d measured using crystal violet. (C) Induction of SOCS3 gene expression depends on nuclear STAT3. mRNA expression of nuclear STAT3 target gene SOCS3 in MDA‐MB‐231 STAT3 WT, KO, KO + WT, and KO + MTS cells. Student’s t‐test: ****= P < 0.0001, ***= P < 0.001, **= P < 0.01, *= P < 0.05, ns = not significant, n = 3. Error bars represent ± SD. Fig. S3. Antioxidant gene expression does not require SIRT1 or SIRT3 activity. (A) mRNA expression of GCLC in MDA‐MB‐231 STAT3 WT or KO cells treated with 200 μm EX‐527 (SIRT1 inhibitor) or 200 μm 3‐TYP (SIRT3 inhibitor) for 24 h. (B) mRNA expression of PGC1α in MDA‐MB‐231 STAT3 WT cells treated with 200 μm EX‐527 or 200 μm 3‐TYP for 24 h. Student’s t‐test: ****= P < 0.0001, ***= P < 0.001, **= P < 0.01, *= P < 0.05, ns = not significant, n = 3. Error bars represent ± SD. Fig. S4. Specificity control for STAT3‐BioID pull‐down. Streptavidin sepharose was used to pull‐down biotinylated proteins from mitochondrial lysates of MDA‐MB‐231 STAT3 WT cells expressing MTS‐BioID treated with 50 μm biotin for 18 h, and complex I components (NDUFV2, NDUFS2, NDUFAF2) were identified by i [file MOL2-15-1432-s002.pdf]

Supplemental Figure S1.

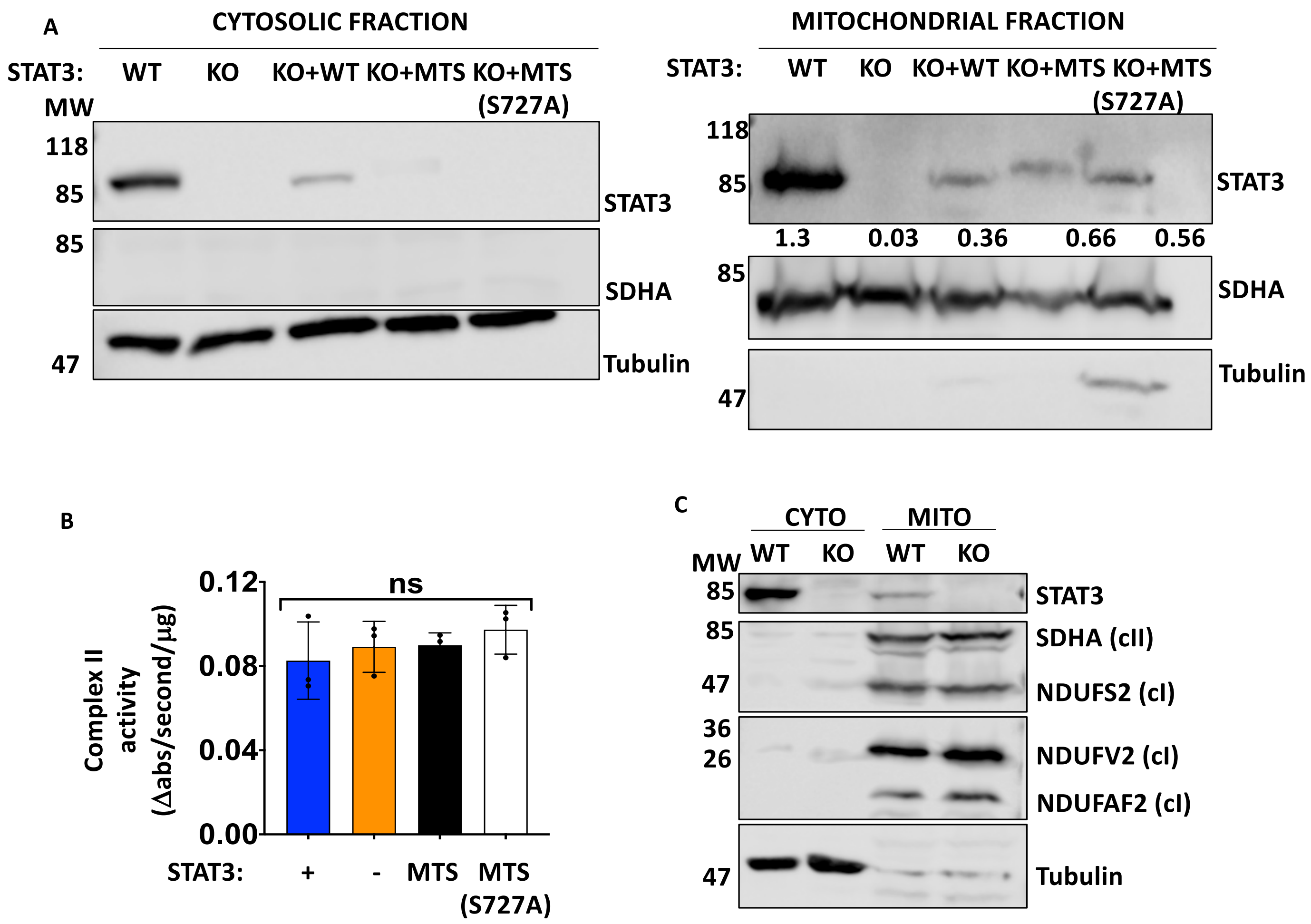

Supplemental Figure S2.

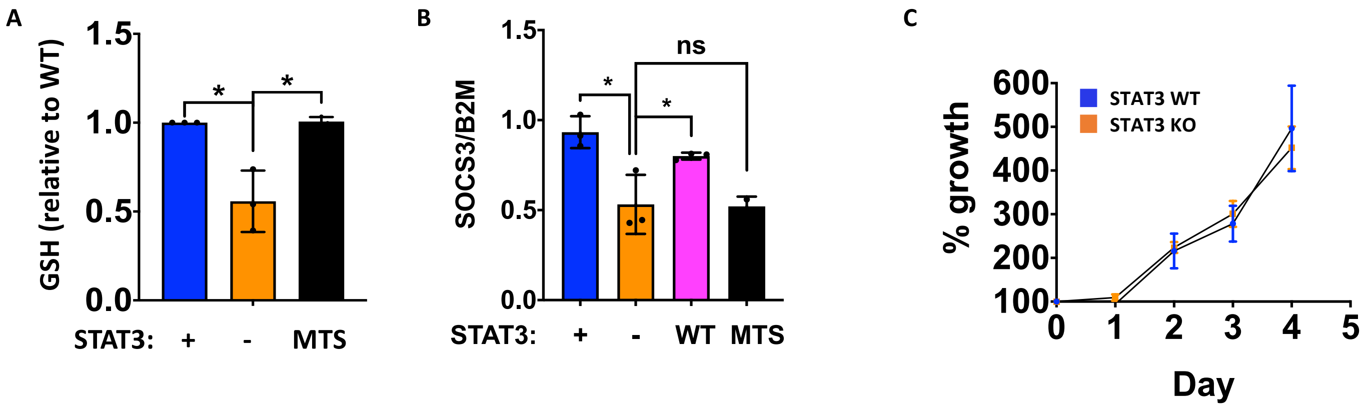

Supplemental Figure S3.

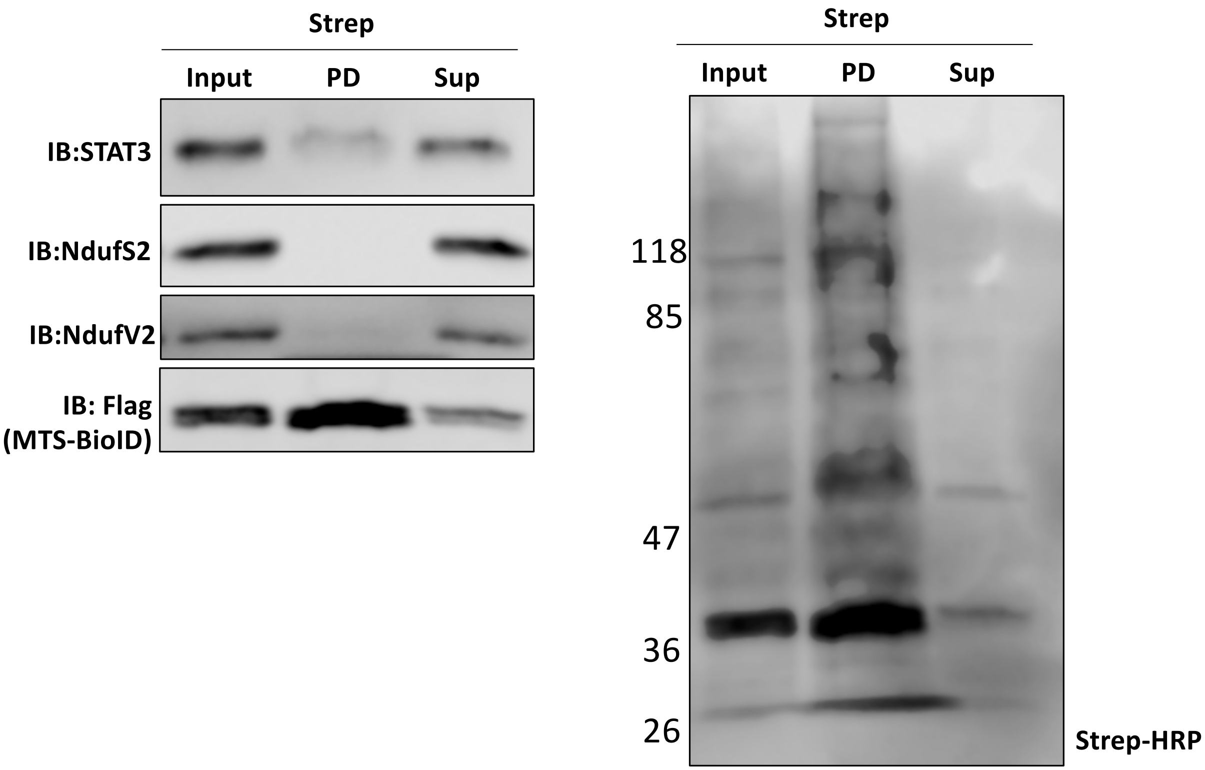

Supplemental Figure S4.

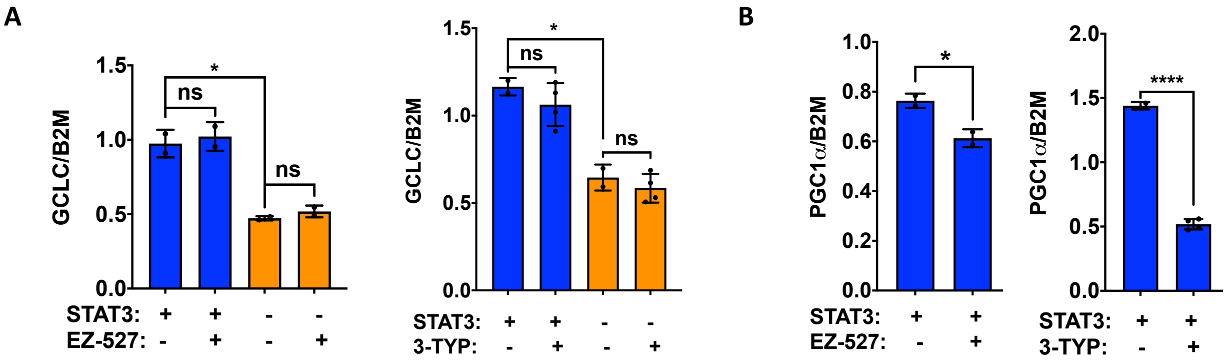

Supplemental Table S1.

Primer Name	Sequence
**GCLC**	F: 5’_AGGCATTGATCATCTCCTGG_3’<br>R: 5_AGGAGGGGGCTTAAATCTCA_3’
**GCLM**	F: 5’_GCTGTATCAGTGGCACAG_3’<br>R: 5_CGCTTGAATGTCAGGAATGC_3’
**NQO1**	F: 5’_GGGCAAGTCCATCCCAACTG_3’<br>R: 5_GCAAGTCAGGGAAGCCTGGA_3’
**HMOX1**	F: 5’_GGCCTGGCCTTCTTCACCTT_3’<br>R: 5_GAGGGGCTCTGGTCCTTGGT_3’
**NFE2LE2**	F: 5’_ ACACGGTCCACAGCTCATC_3’<br>R: 5_TGCCTCCAAAGTATGTCAATCA_3’
**SOCS3**	F: 5’_CCACTCTTCAGCATCTCTGT _3’<br>R: 5_AGCTGGGTGACTTTCTCATA _3’
**PGC1α**	F: 5’_CAAGCCAAACCAACAACTTTATCTCT _3’<br>R: 5_CACACTTAAGGTGCGTTCAATAGTC _3’
**B2M**	F: 5’_TGCTGTCTCCATGTTTGATGTATCT_3’<br>R: 5_TCTCTGCTCCCCACCTCTAAGT_3’
